# The optimal procedure for lateral column lengthening calcaneal osteotomy according to anatomical patterns of the subtalar joint: an anatomical study in the Chinese population

**DOI:** 10.1186/s12891-022-05715-w

**Published:** 2022-08-05

**Authors:** Jiajun Wu, Hua Liu, Can Xu

**Affiliations:** Department of Orthopaedics Surgery, Xiangya Hospital, Central South University, No. 87, Xiangya Road, Changsha, 410008 China

**Keywords:** Subtalar joint, Facet, Anatomic, Lateral column lengthening, Flatfoot, Calcaneal osteotomy

## Abstract

**Background:**

Lateral column lengthening calcaneal osteotomy is a powerful procedure for correcting forefoot abduction in flatfoot deformity. However, it involves the risk of damaging articular facets of the subtalar joint. The optimal method to avoid violating the subtalar joint during lateral column lengthening remained controversial in published reports, implying that the subtalar joint might present anatomical variations among different nationalities. Therefore, the objective of this study was to perform an anatomical study by targeting the healthy Chinese population for the purpose of identifying the optimal procedure for lateral column lengthening calcaneal osteotomy according to anatomical patterns of the subtalar joint.

**Methods:**

A total of 72 ft from 70 fresh frozen cadavers were obtained from the Department of Anatomy of Central South University. For each foot, soft tissues were surgically removed from the bones, and the calcaneus was completely separated from other bones to recognize the anatomical features of the calcaneus. The distance between the calcaneocuboid joint and the articular facet of the subtalar joint was measured by digital calipers for further analysis.

**Results:**

Out of the 72 ft, 36.1% had separated anterior and middle facets in the calcaneus, and 63.8% had partly or completely fused anterior and middle facets. In the calcanei with discrete facets, the mean distance from the calcaneocuboid joint to the proximal margin of the anterior facet was 12.75 ± 2.10 mm, and the mean width of the separation between the anterior and middle facets was 2.43 ± 1.41 mm. In the calcanei with partly or completely fused anterior and middle facets, the mean width of the narrowest part of the tarsal sinus was 5.81 ± 0.62 mm and 6.25 ± 0.35 mm, respectively.

**Conclusions:**

The anatomy of the subtalar joint presents significant individual variations in the Chinese population. Calcanei with partly or completely fused anterior and middle facets were observed in nearly two thirds of individuals. Since the modified Evans procedure might potentially incur damage to the subtalar joint facets, the Hintermann procedure or other modified extra-articular lateral column lengthening procedures may be more applicable to the Chinese population.

## Background

Adult-acquired flatfoot deformity (AAFD), characterized by medial arch collapse, hindfoot valgus, and forefoot abduction [1], is a common clinical problem presenting to foot and ankle surgeons. Among several surgical management approaches for AAFD, the lateral column lengthening (LCL) calcaneal osteotomy is a powerful procedure for correcting forefoot abduction. Evans first described the LCL osteotomy in 1975 [2], which was later modified by Mosca in 1995 [3]. Developed as a joint-sparing procedure, the modified Evans LCL osteotomy was expected to deliver an optimal outcome if it could pass through the interval between the anterior and middle facets of the calcaneus [3]. However, this interval was often difficult to be adequately visualized during operation, so the modified Evans procedure might involve the risk of damaging the articular facets of the subtalar joint. For the purpose of protecting the anterior and middle articular surface, Hintermann proposed a new LCL osteotomy in 1999, which is performed at the anterior border of the posterior subtalar facet [1]. Both the modified Evans and Hintermann procedures have yielded excellent clinical and radiological results [4–8].

The location and direction to perform LCL osteotomy is the key to avoiding violation to the subtalar joint. However, the optimal location and direction for the Evans procedure varied in published studies. Trnka et al. [9] suggested starting the operation at 4 mm proximal from the calcaneocuboid joint, whereas Chan [5], Lombardi [10], and Evans [2] recommended an entry point at 1.5 cm proximal from the calcaneocuboid joint line. The entry point proposed by Mosca was at 1.5-2 cm posterior to the calcaneocuboid joint [3, 11], which was farther than what recommended by Evans. Hyer et al. [12] claimed that the optimal location for LCL osteotomy was at 1.1–1.5 cm (average, 1.3 cm) proximal from the calcaneocuboid joint. Raines [13], Kou [14] and Mosier-LaClair [15] suggested starting the procedure at a position in parallel with and slightly more than 1 cm posterior from the calcaneocuboid joint. Bussewitz [16] recommended an entry point at an angle form the posterior and lateral direction to the anterior and medial direction. The differences in aforementioned studies may be attributed to the anatomic variations among individual patients. Multiple studies have shown that the articular facets of calcaneus exhibit obvious variations across different nationalities.

Bunning and Barnett [17] examined the morphology of the subtalar joint in the European, African, and Indian populations; El-Eishi [18] observed 200 dry calcaneus specimens of Egyptian adults and Jung et al. [19] analyzed the types of subtalar joint facets in the Korean population. The combined results of these studies suggest that each nationality may have some unique features. To date, anatomic studies investigating the anatomical relationship between the articular facet of calcaneus and the LCL are still rare [16], and none of such studies has evaluated the risk of LCL osteotomy in damaging the subtalar joint facets in the Chinese population. The optimal procedure of LCL osteotomy for the Chinese population therefore remains uncertain. In view of this, the objective of this study was to perform an anatomical study by targeting the healthy Chinese population for the purpose of identifying the optimal procedure for LCL calcaneal osteotomy according to anatomical patterns of the subtalar joint.

## Methods

A total of 72 ft from 70 fresh frozen cadavers were acquired from the Department of Anatomy of Central South University. The demographic data including sex, age and foot side was recorded. Only the paired calcanei and tali with complete facets were examined, while variations in other parts were not considered. Specimens involving fractures and/or pathological changes were excluded.

According to morphological features of the subtalar joint facets, the calcanei were classified into three types as follows:

Type A: there are three facets on the surface of calcaneus, with the anterior and middle facets separated from each other;

Type B: there are two facets on the surface of calcaneus, with the anterior and middle facets partly connected;

Type C: there are two facets on the surface of calcaneus, with the anterior facet completely fused with the middle facet.

**Fig. 1:**
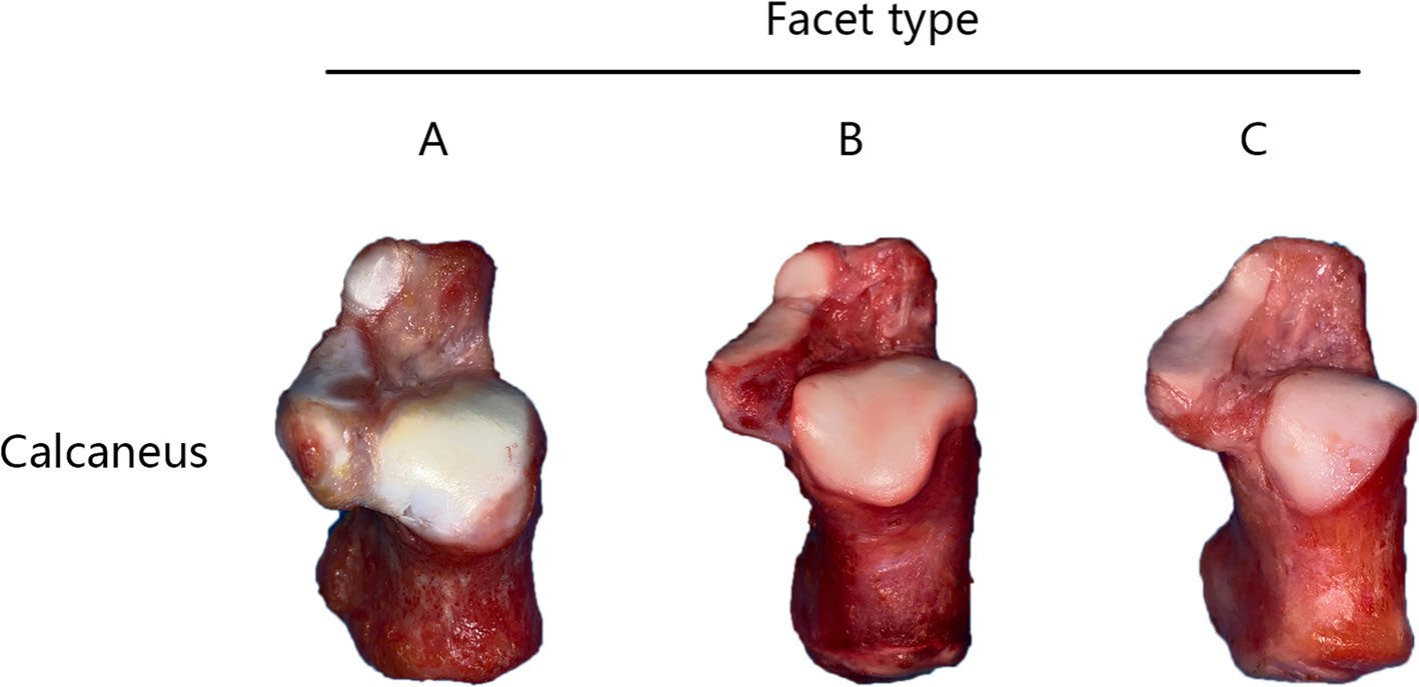
Types of joint facets in the calcaneus

**Fig. 2:**
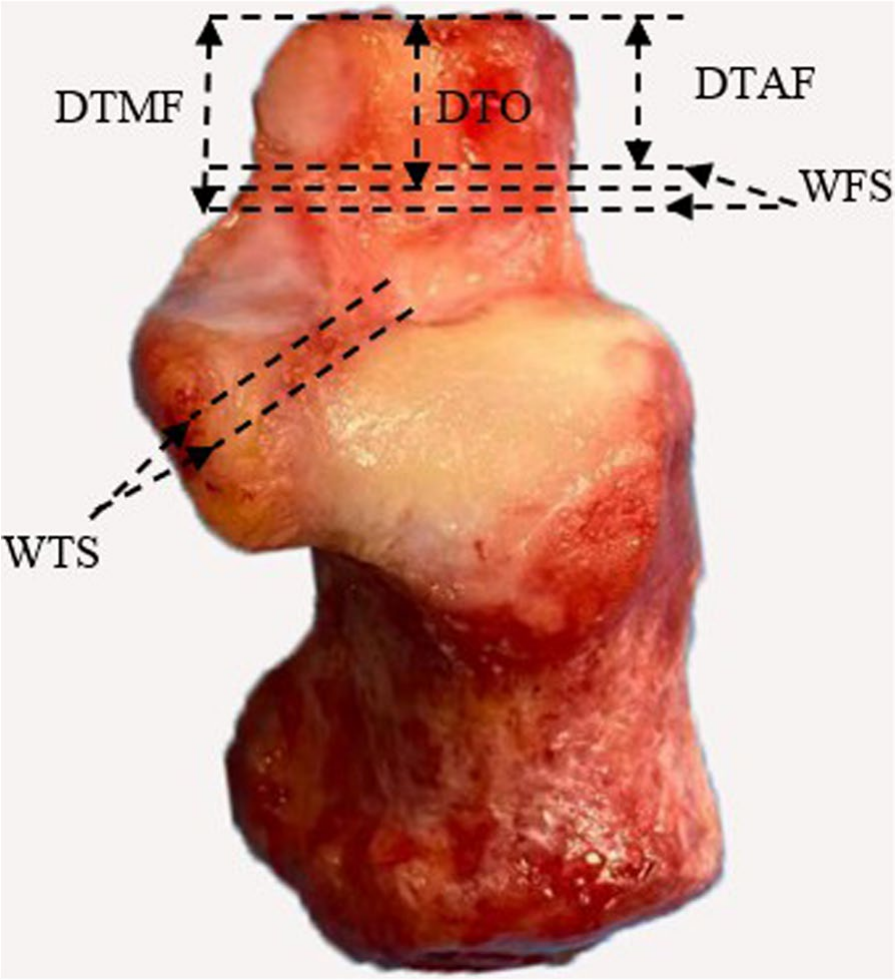
Measurements of the distance. DTAF: The distance from the calcaneocuboid joint to the proximal margin of the anterior facet. DTMF: The distance from the calcaneocuboid joint to the distal margin of the middle facet. WFS: The width of facet separation. WTS: The width of the narrowest part of the tarsal sinus

The specimens were grouped according to the above mentioned classification standard based on naked-eye observation under the aid of a hand lens (Fig. 1), and the distribution was assessed for each type of calcaneus. To reduce variability, the measurements of joint facets were recorded by the same surgeon (L.H.) using digital calipers (Mitutoyo, accuracy 0.01 mm). An intra-rater reliability test was performed, which showed a Kappa coefficient of 0.837. The anatomic parameters were obtained by following the method described by Hyer [12] and Bussewitz [16]. Specifically, for type A calcanei, the distances from the calcaneocuboid joint to the proximal margin of the anterior facet (DTAF) and to the distal margin of the middle facet (DTMF), as well as the width of facet separation (WFS), were measured (Fig. 2). The optimal location of osteotomy was at the midsection of the facet separation. For type B calcanei, the DTAF was measured as the distance from the calcaneocuboid joint to the narrowest portion of the fused facet. For both type B and C calcanei, the width of the narrowest part of the tarsal sinus (WTS) was measured. This interval between the middle and posterior facets was the ideal location for Hintermann LCL osteotomy [1]. The DTAF, DTMF and WFS were all measured in perpendicular to the lateral wall of the calcaneus so as to simulate intra-operative surgical planning [12, 16].

Data was statistically analyzed in SPSS (ver. 26, IBM Institute Inc., Armonk, NC, USA). Continuous variables were calculated as mean ± standard deviation, while categorical data was expressed as counts and percentages. To compare the morphological data between type A, B, and C calcanei, independent sample T-test was performed on quantitative variables.

## Results

The demographic data including sex, age and foot side was presented in Table 1. A total of 72 paired calcanei were examined, involving three types (Table 2). Specifically, 36.1% were classified into type A (all the three facets separated from each other), 19.4% were classified into type B (the anterior and middle facets partly connected), and 44.4% were classified into type C (the anterior facet completely fused with the middle facet).

**Table 1:** The demographic data of acquired specimen

Characteristics		No.	Percentage(%)
Sex	Male	30	42.8
	Female	40	57.1
Side	Right	27	37.5
	Left	45	62.5
Average age (years)		43.5 ± 25.2

**Table 2:** Incidence of different facet types in the calcanei

Bones	Type	No.	Percentage(%)
Calcanei	A	26	36.1
	B	14	19.4
	C	32	44.4

For the measurements of type A calcanei, the mean value of DTAF, WFS and WTS was 12.75 ± 2.10 mm, 2.43 ± 1.41 mmand 4.5 ± 1.87 mm, respectively (Table 3). For type B calcanei, the mean distance from the calcaneocuboid joint to the narrowest portion of the fused facet was 9.75 ± 0.65 mm, and the mean value of WTS was 5.81 ± 0.62 mm. For type C calcanei, the mean value of WTS was 5.81 ± 0.62 mm. It can be seen that the mean WTS of type A calcanei was significantly smaller than that of type B and C (*P* < 0.05, Table 3).

**Table 3:** The mean distance from the calcaneocuboid joint to the subtalar joint facets

Distance	Type A	Type B	Type C	*P* values
DTAF	12.75 ± 2.10	9.75 ± 0.65	/	<0.05
DTMF	16.0 ± 1.22	/	/	
WFS	2.43 ± 1.41	/	/	
WTS	4.5 ± 1.87	5.81 ± 0.62	6.25 ± 0.35	<0.05

DTAF: the distances from the calcaneocuboid joint to the proximal margin of the anterior facet; DTMF: the distances from the calcaneocuboid joint to the distal margin of the middle facet; WFS: the width of facet separation; WTS: the width of the narrowest part of tarsal sinus

## Discussion

The calcanei with partly or completely fused anterior and middle facets (type B + type C) accounted for 63.8% of the total sample. In view of a large interval between the middle and posterior facets, the Hintermann LCL procedure may well adapt to these two types of calcanei. The proportion of calcanei in which a truly extra-articular Evans osteotomy might be suitable was only 36.1% (type A). However, type A calcanei had a smaller interval between the anterior and middle facets (2.43 ± 1.41 mm), making the modified Evans osteotomy difficult to be performed in a truly extra-articular fashion.

LCL osteotomy was first described by Evans in 1975 as a treatment of pediatric flatfoot [2], but later it has become an important option for correcting flatfoot deformity in all age groups. Evans performed the osteotomy at the neck of the calcaneus that was 1.5 cm proximal to and parallel with the calcaneocuboid joint and inserted a trapezoidal wedge of tricortical bone. Later in 1995, Mosca [3, 11] described a modified Evans osteotomy, where the interval between the anterior and middle facets need to be palpated first (usually with a freer elevator). This interval was used to determine the location of osteotomy for the purpose of avoiding damage to the articular facets of the subtalar joint. The procedure was performed at 1.5–2 cm proximal to the calcaneocuboid joint but aiming for the interval between the anterior and middle facets, then the line is slightly oblique from proximal-lateral to distal-medial. However, it is often difficult to identify this interval during the actual operation process because of the limited surgical view obscured by the talus, making anatomic studies on the optimal location of LCL osteotomy very meaningful. There have been numerous anatomic reports modifiying this procedure by changing the distance of the osteotomy from the calcaneocuboid joint or altering the angle of the osteotomy, but little agreement has been reached.

Raines [13] carried out the very first anatomic study on the various structures at risk during the Evans osteotomy. By performing the procedure on 20 cadavers to detect which structures were at risk, he found that the ideal location to avoid damage to the anterior and middle facets was at 10 mm proximal from the calcaneocuboid joint [13]. Hyer et al. [12] conducted an anatomic study with a sample of 768 cases. For type A calcanei, they detected a mean separation of 0.39 cm between the anterior and middle facets, and recommended that the optimal location for Evans osteotomy was at 1.1–1.5 cm (average, 1.3 cm) proximal from the calcaneocuboid joint. However, Bussewitz et al. [16] reported that an entry point in parallel with and at 1.3 cm proximal from the calcaneocuboid joint might also put the facets and sustentaculum tali at risk. The inconsistency across these studies may be attributed to the anatomic variations among individual patients.

The types of calcanei have been investigated in many different nationalities, including Korea [19], India [20], Japan [21], Africa [17], Egypt [18], America [12, 22], Spain [23], and Turkey [24]. Despite differences in the classification methods, the proportions of various types of calcanei in most studies were consistent (Table 4). Specifically, the proportion of separated anterior and middle facets in the total population was mostly ranged from 30 to 40%. Only the Belgium [25] and UK [17] studies reported more type A cases than type B plus type C (67% in UK and 61% in Belgium). In general, more people tend to have connected anterior and middle facets, either partly or completely.

**Table 4:** The types of facets of calcaneus in different nationalities

Study	Country	N	A (%)	B (%)	C (%)	B + C (%)	Others(%)
Bunning [17]	British	194	67.0	/	/	33.0	0.0
Barbaix [25]	Belgium	134	61.0	14.0	14.0	28.0	11.0
Nakashima [21]	Japan	202	49.0	/	/	50.0	1.0
El-Eishi [18]	Egypt	200	40	/	/	49	11
Campos [23]	Spain	176	39.8	29.0	24.4	53.4	6.8
Jung [19]	Korea	118	39.0	32.2	28.8	61.0	0.0
Ragab [22]	America	1056	37	/	/	58	5
Hyer [12]	America	768	41.06	/	/	56.03	2.91
Bunning [17]	Africa	492	36.0	/	/	63.0	1.0
Uygur [24]	Turkey	221	34.4	25.3	33.0	58.4	7.2
Gupta [26]	India	401	25.9	28.0	39.0	66.8	7.2
Present study	China	72	36.1	19.4	44.4	63.8	0.0

Similarly, it was also found in our study that the calcanei with partly or completely fused anterior and middle facets (type B + type C) accounted for a major proportion in the Chinese population. For these two types of calcanei, the anterior and middle facets are fused as a large anterior facet, which will be inevitably violated during the modified Evans osteotomy. Therefore, the Hintermann LCL procedure might be a better option. In particular, based on our observation, both type B and type C calcanei had a large interval between the middle and posterior facets, which could provide an ideal entry point for the Hintermann osteotomy.

However, it was also revealed by our study that to perform a truly extra-articular modified Evans osteotomy was difficult for type A calcanei in the Chinese population. Since the mean width between the anterior and middle facets is only 2.43 mm, in order to achieve an extra-articular osteotomy for type A calcanei, the surgeon may have to correctly find a 2 mm wide path at an indeterminate trajectory with a saw blade that is typically around 1 mm wide. This seems to be a nearly impossible mission. Contrary to the Chinese population, Hyer et al. [12] detected a mean width of 0.39 cm between the anterior and middle facets in the American population, making the modified Evans osteotomy feasible. Therefore, Chinese individuals with type A calcanei may be exposed to a higher risk of damaging the anterior and middle facets of the subtalar joint during the operation, making the Hintermann osteotomy a potentially better option to protect their anterior and middle facets. Unfortunately, the WTS of Type A calcanei was significantly smaller than that of Type B and C calcanei. The mean width between the middle and posterior facets was 4.5 mm, and in some specimens, this interval was only 2.3 mm wide, which made the Hintermann osteotomy extremely difficult to perform. For calcanei involving such special situations, other modified procedures described in recent years that preserve the subtalar joint in its entirety (e.g., extended Z-cut osteotomy [27] and modified extra-articular LCL [28]) may be more applicable.

The Hintermann and modified Evans procedures have been compared with each other from multiple perspectives [29–31]. Ettinger compared the anatomic structures at risk during the modified Evans osteotomy versus the Hintermann osteotomy [29] and reported that the Hintermann osteotomy was a superior option in terms of the potential damage incurred to the articular surfaces of the subtalar joint. Koury compared the biomechanical effects between the modified Evans osteotomy and Hintermann osteotomy [31], and the results suggested that the Hintermann osteotomy could minimize the increase in calcaneocuboid joint pressure and reduce the risk of subsequent calcaneocuboid osteoarthritis. Several studies focused on the clinical outcomes after the modified Evans osteotomy and Hintermann osteotomy and reported good clinical and radiographic results for both procedures [4–8]. Moreover, a direct clinical comparative study of outcomes between the modified Evans and Hintermann procedures showed comparable results between the two [30], but the calcaneocuboid joint tended to develop fewer degenerative changes after the Hintermann procedure. In general, with a lower risk to the subtalar and calcaneocuboid joints but similar clinical outcomes, the Hintermann procedure seems to be a better alternative to replace the Evans osteotomy in the Chinese population. Other novel extra-articular LCL procedures [27, 28] may also be applicable to the Chinese population, but more extensive clinical observations are required.

It was sincerely acknowledged that our study was subjected to several limitations. First, our specimens were acquired from the normal population, but LCL osteotomy is only performed on flatfoot patients in clinical practice. Therefore, the results would have been more meaningful if our study was based on flatfoot specimens. Nevertheless, previous studies implied that type B and type C together might occupy a dominant proportion in the AAFD population [32], and these two patterns had been carefully analyzed in our study. Second, due to limited sources of fresh human specimens, our sample size (72 ft) might not be able to fully represent the general situation in the Chinese population. A bone bank [12] or numerical (3D CT) [33] study may help generate more samples. However, since the cartilage in dry calcaneus has been lost and CT reconstruction is not able to accurately reproduce the cartilage, it might lead to errors in measuring the distances such as DTAF, DTMF, WFS and WTS. Third, the curative effect of the LCL procedure depends on many factors including correct indication, appropriate concomitant procedures, use of stabilizing pins and bone graft, so although the Hintermann procedure has a lower risk to the subtalar joint, further observations are needed before reaching a conclusion on its superiority. At last, recent developments in the medical imaging technology provide possibilities to perform pre-operative surgical planning using non-invasive and 3D techniques such as radiostereometry, MRI and 3D printing technology, which may all be valuable tools to determine the optimal procedures to be performed.

## Conclusions

In conclusion, the anatomy of the subtalar joint exhibited significant individual variations in the Chinese population. Calcanei with partly or completely fused anterior and middle facets were observed in nearly two thirds of individuals. Since the modified Evans procedure might potentially incur damage to the subtalar joint facets, the Hintermann procedure or other modified extra-articular LCL procedures may be more applicable to the Chinese population.

## Data Availability

The datasets used and analyzed during the current study are available from the corresponding author on reasonable request.

## Abbreviations

AAFD: Adult-Acquired Flatfoot Deformity
LCL: Lateral Column Lengthening
DTAF: The Distances from the Calcaneocuboid joint to the proximal margin of the Anterior Facet
DTMF: The Distances from the Calcaneocuboid joint to the distal margin of the Middle Facet
WFS: The Width of Facet Separation
WTS: The Width of the narrowest part of Tarsal Sinus
